# Supplementary material for: Novel Driver Strength Index highlights important cancer genes in TCGA PanCanAtlas patients
Source: PeerJ. 2022 Aug 11;10:e13860. doi: 10.7717/peerj.13860 (PMC9375969; doi:10.7717/peerj.13860)
Supplement: Supplemental Information 2 [file peerj-10-13860-s002.zip › COHORTS/genes plots/age/2021_8_16_14_9_distribution_cohorts_females_25-29.pdf]

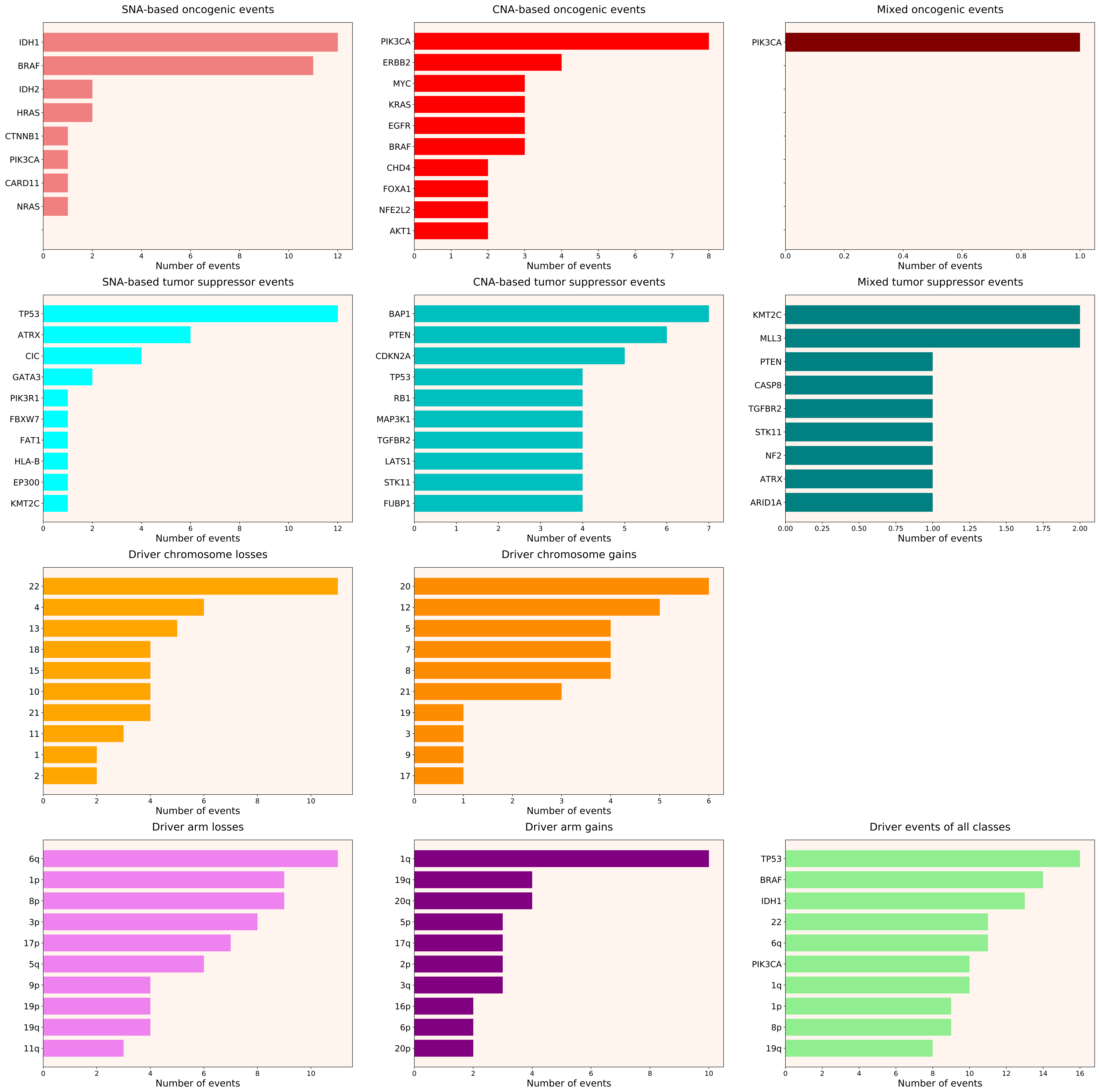
SNA-based oncogenic events
IDH1
BRAF
IDH2
HRAS
CTNNB1
PIK3CA
CARD11
NRAS
Number of events
CNA-based oncogenic events
PIK3CA
ERBB2
MYC
KRAS
EGFR
BRAF
CHD4
FOXA1
NFE2L2
AKT1
Number of events
Mixed oncogenic events
PIK3CA
Number of events
SNA-based tumor suppressor events
TP53
ATRX
CIC
GATA3
PIK3R1
FBXW7
FAT1
HLA-B
EP300
KMT2C
Number of events
CNA-based tumor suppressor events
BAP1
PTEN
CDKN2A
TP53
RB1
MAP3K1
TGFBR2
LATS1
STK11
FUBP1
Number of events
Mixed tumor suppressor events
KMT2C
MLL3
PTEN
CASP8
TGFBR2
STK11
NF2
ATRX
ARID1A
Number of events
Driver chromosome losses
Number of events
Driver chromosome gains
Number of events
Driver arm losses
Number of events
Driver arm gains
Number of events
Driver events of all classes
TP53
BRAF
IDH1
PIK3CA
Number of events
